# Evaluation on the Nutrition Quotient Scores of Elderly People Living Alone in Korea

**DOI:** 10.3390/nu15173750

**Published:** 2023-08-27

**Authors:** Gyoungok Gang, Min June Lee, Eun-hui Choi, Hye-Lim Lee, Hyun-Young Lee, Hye-Ja Chang, Jung-Hwa Choi, Na-Young Yi, Kyung-Eun Lee, Min-Jae Chung, Tong-Kyung Kwak

**Affiliations:** 1 Department of Food & Nutrition, Yonsei University, 50 Yonsei-ro, Seodaemun-gu, Seoul 03722, Republic of Korea; 2 Department of Food Science & Nutrition, Dankook University, 119, Dandae-ro, Dongnam-gu, Cheonan-si 31116, Republic of Korea; 3 Department of Food & Nutrition, Soongeui Women’s College, 10 Sopa-ro 2-gil, Jung-gu, Seoul 04628, Republic of Korea; 4 Department of Food & Nutrition, Daejeon University, 62 Daehak-ro, Dong-gu, Daejeon 34520, Republic of Korea; 5 Division of Applied Food System, Seoul Women’s University, 621 Hwarangro, Nowon-gu, Seoul 01797, Republic of Korea; 6 Department of Food & Nutrition, Shingu College, 337 Gwangmyeong-ro, Jungwon-gu, Seongnam-si 13174, Republic of Korea

**Keywords:** nutrition quotient for elderly, elderly living alone, food behavior, nutritional status

## Abstract

As the Korean society is aging rapidly, the issues on physical, social, economic, and mental disabilities of single-person households aged 65 years or older has also increased. This study aimed to investigate the nutrition-related dietary conditions of elderly people living alone and determine their dietary behavior by calculating the nutrition quotient for elderly (NQ-E). One hundred and three elderly people living alone who were basic living recipients were recruited from six senior welfare centers in Seoul, and the data were collected using a questionnaire from 19 July 2016 to 17 August 2016, with a 1:1 in-depth interview using the modified version of the NQ-E questionnaire. The data were analyzed using SPSS 27.0 for Mac (IBM Corp., Armonk, NY, USA); a *p* value of <0.05 was considered significant. The nutrition-related dietary conditions of the elderly living alone were limited, and many of them received support from the government, which helped improve their diet. The nutrition quotient score of the elderly living alone was 50.14, which was lower than the NQ-E mean score (57.6) of the Korean elderly and the NQ-E (62 points), which is the top 25% of the national survey subjects according to the criteria value presented by the Korean Nutrition Society. Elderly people living alone often have poor dietary habits and nutritional status. The NQ-E presented in this study can be used to evaluate the dietary conditions of the elderly and is expected to be used as an indicator for developing community programs for health promotion and evaluating their effectiveness.

## 1. Introduction

An increase in the elderly population is a global phenomenon. As of 2022, the global composition percentage of the population aged 65 years or older was 10.3% [1]. Korea’s elderly population reached 17.5% in 2022 and will increase to 20.6% in 2025; an increase in this population will turn Korea into a super-aged society by 2025 [2,3]. This rapid change can be attributed to Korea’s high life expectancy of 83.5 years in 2020, which was only 62.1 years during the early 1970s [1]. As such, the nation’s aging population is increasing at a very rapid pace.

The rapid change in the population composition brought about the changes in household types. In Korea, the percentage of elderly households was 22.4% in 2020. This percentage is expected to increase to 49.8% in 2050 [3,4]. In particular, single-person households aged 70 years or older are expected to occupy the highest share at 42.9% in 2050, while the ratio of the four households is decreasing gradually [4]. The number of elderly households living alone has increased since 2010, which is consistent with a global trend [3]. Due to the change in the family type of senior populations and the increase in the number of senior populations, both elderly people living alone and the elderly who are married are the most prominent family types.

Old age is the period when physical, social, economic, and mental abilities decrease an individual’s participation in family and social activities [5,6,7], which significantly affects the elderly’s lifestyle [8]. During old age, single-person households generally encounter economic instability and challenges such as a decrease in income [5,9,10]. A multitude of factors contribute to the nutritional deficiencies of elderly people, such as low income and education levels, poor dental health, decreased vision and hearing, isolation and depression, cognitive decline, difficulty in grocery shopping and cooking, and the intake of certain medications [8,11]. They also experience physical limitations and functional decline due to the aging processes and presence of chronic diseases, which in turn causes loss of appetite and desire for life [12,13]. Therefore, since they are exposed to many risk factors that hinder healthy dietary intake, they easily develop nutritional imbalance and require special nutritional needs [3]. 

In general, elderly people who live alone have more difficulties in managing health than the elderly with families [14]. Elderly people living alone have become an essential subject for food purchase and diet management in single-person households, and they usually face issues related to the quality and quantity of their diet, and lack overall energy and major nutrients, making them vulnerable to malnutrition; hence, they need diet management to improve these factors [15,16]. Those living alone have higher incidence of chronic diseases, falls, nutritional imbalance, depression, and suicide than elderly couples or elderly couple households with children cohabitation [2,9]. Therefore, provision of healthcare and implementation of preventive measures through diet management and elderly nutrition should lead to healthy aging. Such healthy aging improves the quality of life of elderly people and reduces the burden of soaring medical costs, thus enhancing the overall economic efficiency [8].

As elderly people living alone experience severe economic, physical, and mental problems and social isolation [5], the government and local governments have begun to devise various support systems that have started to provide the needs of elderly people living alone and help improve their overall quality of life [17]. The Seoul Metropolitan Government currently provides care services for elderly people living alone, such as food delivery, side dish deliveries, and meal plans [9]. A study of food support services by type of residence shows that the elderly people in single-person households use more meal support services, 2.04% in cohabitation households, and 10.42% in single-person households [9].

In addition, various indicators have been developed and used to evaluate the dietary behavior and nutritional status of the elderly. In the United States, the Nutrition Screening Initiative was developed to identify and treat nutritional problems in the elderly [18]. The Dietary Quality Index was developed in 1994 and consists of 10 items (100 points) related to food and nutrients, which can measure the diet quality [19]. In 1995, the United States Department of Agriculture (USDA) developed the Healthy Eating Index (HEI) based on a 10-component system of five food groups, four nutrients, and a measure of variety in food intake [20]. The Alternate Healthy Eating Index was a more comprehensive tool for predicting chronic disease risk than the HEI, which claimed that dietary guidelines could be improved by providing more specific and comprehensive advice [21].

Putting these studies together, it is necessary to analyze the dietary status, behavior, and nutritional status of the Korean elderly, especially elderly people living alone. This study aimed to investigate the nutrition-related dietary conditions of elderly people living alone and to determine their dietary habits by calculating the nutritional quotient. The current status of dietary support, dietary conditions, and dietary behavior was investigated to determine the dietary life of elderly people living alone. Based on this, the nutrition quotient for each factor of elderly people living alone was calculated using the nutritional quotient model for dietary life management across the life span developed by the Korean Nutrition Society [22] and compared with the nutrition quotient for elderly (NQ-E) of the Korean elderly. The results of this study can be used as a basis for improving the general dietary habits and nutritional management ability of elderly people living alone. It can also be used as an institutional basis for implementing dietary and nutritional management for the elderly.

## 2. Materials and Methods

### 2.1. Data Collection

A total of 103 elderly people living alone and in low-income households participated through a convenient sampling method in Seoul, excluding those who answered the questionnaire poorly. They received a “basic care service for the elderly” to be eligible for the survey. The basic care service for the elderly is a public service provided free of charge to seniors aged 65 or older who live alone, need regular safety checks, or need welfare services due to difficulties in living. It mainly provides health status, regular safety checks, exercise and nutrition management, life education, and life support. A senior life manager visits weekly, checks the phone 2–3 times, and conducts life education twice a month. On the other hand, basic living recipients refer to those whose household income is less than 30–50% of the standard median income. Under the National Basic Living Security Act, they receive support for living expenses of ‘living benefit, medical benefit, housing benefit, and education benefit’. The survey was conducted from 19 July 2016 to 17 August 2016, with a 1:1 in-depth interview using the developed questionnaire at six senior welfare centers in Seoul or participants’ houses. This study was approved by the Yonsei University Bioethics Review Board (IRB 1040917-201607-S8-204-03).

### 2.2. Survey Protocols

Data on the participants’ gender and age were obtained, and their economic and educational levels were examined based on their social and demographic characteristics. An interview survey was conducted using the nutrition quotient for Korean elderly (NQ-E). 

The NQ-E is a questionnaire developed to evaluate the dietary quality and nutritional status of the elderly in Korea by the Korean Nutrition Society [7]. The NQ-E questionnaire consists of 19 items in four factors: ‘food behavior’, ‘balance’, ‘diversity’, and ‘moderation’.

In the ‘food behavior’ factor, there are six items that affect the eating behavior of the elderly, such as the degree of discomfort in chewing food, degree of depression, awareness of health, efforts to have a healthy diet, hand washing before meals, and daily exercise time. The ‘balance’ factor consisted of four items: frequency of consumption of milk and dairy products, fruit, snacks, and water. The ‘diversity’ factor included six items: frequency of consumption of eggs, fish or shellfish, vegetables, soybeans or soy products, number of meals per day, and frequency of eating alone. In the ‘moderation’ factor, the foods that the elderly tend to consume excessively are composed of three items: frequency of consumption of sweet or greasy bread, sweetened beverages, and ramen noodles.

The NQ-E score was finally calculated after adding the weighted scores for each question and applying statistically calculated weights for each factor (food behavior 0.3, balance 0.2, diversity 0.2, moderation 0.3). If the final summed score of subjects in this study is 62 points or more, which is in the top 25% of national survey subjects based on mean nutrient adequacy ratio (MAR), it is classified as ‘good’, and if it is less than 62 points, it is classified as ‘monitoring needed’ [7]. In this preceding study, the construct validity of the NQ-E was evaluated using confirmatory factor analysis, LISREL.

NQ-E = balance × 0.20 + moderation × 0.30 + diverse × 0.20 + food behavior × 0.30

### 2.3. Statistical Analysis

The data were analyzed using SPSS 27.0 for Mac (IBM Corp., Armonk, NY, USA). Descriptive statistics were computed for all variables. For food insecurity and social activity, nutrition status and dietary life, dietary behaviors, frequencies and percentages were calculated by χ^2^ test or Fisher’s exact test. An independent *t*-test was performed to compare the nutritional quotient values by gender. All statistical analyses were performed with a significance level of *p* < 0.05.

## 3. Results

### 3.1. Respondents’ General Characteristics

The general information of the participants is presented in Table 1. In terms of gender, 26 (25.2%) men and 77 (74.8%) women responded to the survey. A total of 26 (25.2%) individuals aged 65–74 years and 77 (74.8%) individuals aged 75 years or older responded to the survey, and the average age of this group was 77.8. As regards the education level, 42 (40.7%) respondents had no educational experience, 27 (26.2%) were elementary school graduates, 17 (16.5%) were middle school graduates, 9 (8.7%) were high school graduates, 6 (6.8%) were college graduates or higher. 

Concerning the basic living beneficiaries, 62 (60.2%) respondents were basic living recipients, 6 (5.8%) were from the secondary poor, and 28 (27.2%) were not eligible.

The elderly were taking drugs in the order of hypertension drugs (53.4%), diabetes drugs (25.2%), arthritis drugs (22.3%), hyperlipidemia drugs (18.4%), gastrointestinal drugs (13.6%), and anemia drugs (2.9%). In particular, 53.4% of hypertension treatments were taken by more than half of the respondents. Other opinions also confirmed to be taking painkillers, depression drugs, prostate drugs, sleeping pills, calcium supplements, thyroid drugs, and asthma drugs were taken.

### 3.2. Status of Government Support Related to Health and Diet

According to a survey on the health and dietary support status of elderly people living alone by the government or local governments, 102 (99%) people received a visiting service of senior life managers (Table 2). In addition, 55 (53.4%) people received congregate meal service, 18 (17.5%) received side dish delivery services, 13 (12.6%) received foodbank tickets (vouchers), four (3.9%) received lunch box delivery services, and one (1.3%) received rice aid. This study showed that elderly men living alone received 16.0% more free access to senior restaurants and 12.6% more side dish delivery services than elderly women living alone. 

### 3.3. Food Insecurity and Social Activity of Elderly People Living Alone

The survey results on food insecurity of elderly people living alone over the past year indicated that only 25.2% ate enough and different types of food as they wanted (Table 3). Furthermore, this demonstrated that elderly men (38.5%) have better dietary situations than elderly women (20.8%). Approximately, 42.7% of them ate enough food but could not eat various kinds of food, while 32.0% said they lacked food due to financial difficulties. Of the total respondents, 34.0% of the recipients reported that they often engage in social activities, including leisure activities; 10.7% said that they engage in social activities on a regular basis; 39.8% said they do not do much, and 15.5% do not at all engage in social activities. 

### 3.4. Nutrition and Dietary Life of Elderly People Living Alone

This study confirmed that 77.7% of elderly people living alone had breakfast, 95.1% had lunch, and 90.3% had dinner, indicating no significant difference men and women (Table 4). The main reasons for skipping meals were significantly different between men and women. Women pointed to a lack of appetite (15.6%), usual habits (7.8%), poor digestion (5.2%) while men pointed to difficulty in preparing meals (11.5%), and to save money (7.7%). In terms of dietary life problems, 47.6% responded that they had no particular dietary issues, followed by poor appetite (16.5%), unbalanced diet (15.5%), eating disorders due to dental problems (14.6%), eating salty food (10.7%), skipping meals (5.8%), eating irregular meals (4.9%), and eating sweets (4.9%). 

### 3.5. Dietary Behavior of Elderly People Living Alone

Elderly people living alone consumed one (29.1%) or two (33.0%) vegetable side dishes except for kimchi at each meal (Table 5). On the contrary, only 2.9% of participants ate more than four side dishes. Regarding water intake, 45.6% of the respondents said they drank water three to five times a day, and 21.4% drank it six to seven times a day. For the number of meals per day, 62.1% of the respondents said that they ate more than three times a day, 34.0% said they ate twice a day, and 3.9% ate only once. Seventy-three percentage (73.1%) of elderly men had three or more meals per day, while 58.4% of elderly women had the same. 

Additionally, 19.4% of the respondents ate alone daily, 54.4% ate alone twice a day, and 22.3% ate alone once a day. In terms of the number of snacks consumption, 70.9% of elderly people living alone said they did not eat snacks, while only 4.9% of the elderly ate snacks more than twice a day. Of the elderly people living alone, 64.1% tried to eat healthy daily, while 21.4% did not, indicating that elderly people living alone were willing to practice consuming a healthy diet. 

Currently, 49.5% of elderly people living alone have difficulty chewing food due to missing teeth, denture problems, and gum problems, while more than 41.7% do not experience difficulties chewing food. Approximately 92.2% of elderly people living alone maintained hand hygiene, while only 5.8% did not. Of the elderly people living alone, 58.3% performed exercise 1 h a day, 23.3% performed exercise more than 1 h a day, and 18.4% rarely performed exercise. 38.4% of elderly men exercised more than 1 h a day, while 18.2% of elderly women did. 

According to the depression experience, 46.5% said that they feel often or very often depressed. By contrast, 36.9% said that they did not feel depressed, indicating that more elderly people were depressed among those who lived alone. About 50.5% of elderly people living alone thought they were healthy, while 38.8% thought they were unhealthy. Not presented in Table 5, the percentage of people drinking alcohol was approximately 20%, indicating that they usually drink soju.

### 3.6. NQ-E Comparison of Elderly People Living Alone

The nutrition quotient of elderly people living alone was calculated using the NQ-E developed by the Korean Nutrition Society [7,22]. The nutritional quotient was divided into four factors: balance, moderation, diversity, and dietary behavior; the values for each element were calculated. NQ-E for the elderly living alone was 50.14, 50.89 for elderly men, and 49.88 for elderly women (Table 6). The nutrition quotient criteria for elderly people is 62.0 points [22], so NQ-E for the elderly living alone scored very poorly.

Among the four factors, elderly people living alone scored the lowest value on diversity (35.12 points). The balance factor was also low at 37.96 points. However, elderly people living alone scored 64.16 points for moderation (60.94 for men, 65.24 for women) and 54.25 points for dietary behavior (59.36 for men, 52.53 for women), which were relatively high compared with those for balance or diversity of elderly people living alone. But there was not a significant difference between men and women. 

### 3.7. Distribution of NQ-E for Elderly People Living Alone

The NQ-E score distribution of elderly people living alone was analyzed by the NQ-E grade criteria following the study by the Korean Nutrition Society [7,22]. Values are presented in Table 7. The grade criteria represent the range of grade scores classified into quartiles of the NQ-E of the national survey subjects. By applying the percentile value of the survey subjects, it was classified into high (75 ~ 100%), medium-high (50 ≤ 75%), medium-low (25 ≤ 50%), and low (0 ≤ 25%) grades. Only 8.7% were included as high for NQ-E, while 54.4% were low. For balance and dietary behavior factors, 27.2% each were as high. There were none as high for moderation, while 52.4% were low. Only 1.9% were as high for diversity, while 65.0% were low. In this study, NQ-E, moderation, and diversity factors tended to be low. 

## 4. Discussion

This study investigated nutrition-related dietary conditions, dietary support, and dietary behavior. It examined dietary habits by calculating the NQ-E to determine the dietary life of elderly people living alone. Each factor of the NQ-E for elderly people living alone was calculated using the NQ-E model for dietary life management across the life span developed by the Korean Nutrition Society [7]. The NQ-E scores of elderly people living alone in this study were compared with those of the Korean Nutrition Society [22].

The significant findings of the study are as follows:

Elderly people living alone perceived their current health status as positive, but their actual dietary habits and nutritional status was poor.
Elderly people living alone had poor dietary conditions due to their family structure and relatively low social activity, and many received support from the government or local governments, which helped improve their diet.
The NQ-E of elderly people living alone (50.14 points) was much lower than the NQ-E criteria (62.0 points) established by the Korean Nutrition Society or the NQ-E of the Korean elderly (57.6 points), making it essential to improve the diet and nutritional status of this elderly group.

Elderly people living alone had inadequate food intake and poor dietary conditions. Although most elderly people living alone receive one or more healthcare and dietary supports from the government or local governments, they still face economic difficulties, making it hard for them to eat or eat a wide variety of food. The results showed that most elderly single-person households faced economic, health, residential, and social difficulties [5,16]. Food cost is a problem for many elderly people living alone, and economic situations could explain decreased food consumption [14,23]. In many cases, Korean elderly people experience absolute poverty and have difficulty preparing meals due to health problems and financial difficulties [24]. Elderly people living alone also find it challenging to prepare meals three times a day; hence, they usually eat two to three times a day. 

However, the free meals provided to elderly people in the vulnerable class significantly promote their health by improving their nutritional status and supplying major nutrients, which maintain their quality of life [24]. The elderly who received the free meal service had a better nutritional intake than those who did not; the free meal service plays an effective role in improving the nutritional intake of elderly people in the vulnerable class [25]. The free meal service not only encourages the elderly to consume a well-balanced diet but also has a positive effect on them [24]. Thus, social and government policies and support for elderly people living alone must be continuously expanded [5].

Elderly people living alone eat meals alone most of the day, which is attributed to their family structure and low participation in social activities. One in every five elderly people living alone eats alone three times a day, which is far below the Nutrition Society’s criteria for “rarely eating alone.” Elderly men living with a spouse had better dietary patterns than those living alone, and those living alone were at the highest risk of inadequate dietary intake [26]. Those particularly aged 75 years or older consumed a poor-quality diet than men living with a spouse. Due to a lack of cooking skills and the difficulty of preparing meals, men living alone were more often at a greater risk of inadequate dietary intake than women [14]. This study also showed that the proportion of men aged below 79 years who received free lunch service was exceptionally high.

This study confirmed that elderly people living alone experience loss of appetite or have a habit. Studies [16,27,28] showed that elderly people living alone experience a loss of appetite and have a greater malnutrition risk compared to others who cohabit with someone; hence, social isolation is a significant predictor of malnutrition in the elderly. Therefore, the diet of the elderly has been significantly affected by family composition; in elderly people living alone, the intake and quality of nutrients are also extremely low [23,26].

In the Sixth National Health and Nutrition Survey, 9.3% of elderly living alone engage in morning fasting, higher than the 3.5% rate of elderly people with spouses [26]. Since the rate of morning fasting of elderly people living alone in this study was 22.3%, which is significantly higher than that reported in the 2013 National Health and Nutrition Survey [26], it is necessary to develop measures to improve the breakfast rate of elderly people living alone and provide social and institutional support. According to Oh’s study [23], elderly people living alone usually ate lunch and dinner. However, they skipped breakfast, especially male elderly people living alone (9.9%), and this study demonstrated that 26.9% of elderly men living alone also skipped breakfast. 

Many previous studies have demonstrated nutritional deficiencies in the elderly [27]. In people of old age, their special nutritional needs are usually brought up because physical restrictions and functional degradation due to the aging processes and chronic diseases can be accompanied by a decreased appetite for life [12,27]. According to Lee et al. [15], this malnutrition problem is a unique characteristic of the elderly, as older adults lack overall nutrient intake regardless of their income levels compared with other age groups. However, a low-income level in the elderly is associated with a higher risk of developing malnutrition and poorer nutritional status and is related to higher depressive symptoms [16,29]. Kim et al. [30] claimed that most elderly people found it challenging to obtain the proper meal because of financial and physical difficulties. Social, economic, physiological, psychological, and health problems affect older people’s eating habits and dietary intake [31]. Therefore, it is important to determine the general daily intake rather than the intake of a particular nutrient to understand the nutritional status of the elderly [32]. 

About half of the elderly people living alone in this study thought that they did not have any particular nutritional or dietary problems and were healthy. It was also judged that they were trying to consume a healthy diet and were willing to maintain a healthy lifestyle, such as exercising 1 h a day. Nevertheless, the NQ-E of elderly people living alone was calculated using the nutrition quotients across the life span developed by the Korean Nutrition Society in 2015 [22]. The NQ-E of elderly people living alone was extremely low (50.14 points). The NQ-E of the elderly living alone was significantly lower than the NQ-E criteria for Korean elderly (62 points) established by the Korean Nutrition Society and the NQ-E of Korean elderly (57.64 points) investigated in previous studies [7]. The number of elderly people living alone was only 10 (9.7%), which exceeded the NQ-E criteria (62.0 points), while the remaining 93 (90.3%) were unable to achieve the NQ-E criteria. Since the criteria for determining the NQ-E for the Korean elderly were calculated by the Korean Nutrition Society based on a score of 62 points from the top 25% of the nation’s survey participants [7,22], it was found that the proportion of elderly people living alone with good NQ-E score was relatively low. Studies [33,34] showed that NQ-E is an effective measurement to evaluate the dietary life and nutrition status of elderly people. Therefore, monitoring the NQ-E score of elderly people living alone is necessary to improve their diet and nutritional status. 

In terms of the four NQ-E factors, elderly people living alone obtained the lowest score in the diversity factor (35.12 points); therefore, the elderly need to avoid eating alone, improve the number of meals consumed per day, and eat a variety of food including eggs, fish or shellfish, vegetables, and soy or soybean products. As shown in Ham’s study [33], this finding indicates the lack of diversity in the NQ-E scores of elderly people in Seoul. The low value in the diversity and balance factors of elderly people living alone indicated the same tendency as the low score in these parts of the nutrition quotient for the Korean elderly, according to the Korean Nutrition Society [7]. However, Gham [35] reported that the scores of the elderly people in Gyeonggi-do Province for the four NQ-E factors, dietary behavior, diversity, balance, and moderation, were higher than the NQ-E criteria; therefore, they seemed to have relatively good health. Only elderly women who did not eat healthy, functional foods scored lower on diversity. Elderly people living alone also scored lower on balance (37.96), and it is believed that milk or dairy, fruit, snack, and water intakes as the items evaluated under the balance factor, were insufficient. 

Among the NQ-E factors of elderly people living alone, the moderation factor score was 64.16 points. The dietary behavior factor score was 54.25 points, which was relatively high compared with the balance or diversity factor scores. However, these scores are still lower than those factors of the NQ-E criteria, 76.49 points on moderation and 55.00 points on dietary behavior by the Korean Nutrition Society [7,22]. The challenges in acquiring and preparing food contributed to the limited diversity of foods consumed by elderly people living alone; hence, elderly people living alone need to have an adequate intake of core foods, especially fruits, vegetables, and fish [14].

The low NQ-E scores for elderly people are attributed to the difficulty of taking nutrients due to loss of body function, tooth loss due to aging, and poor digestion. Thus, this negatively affects the dietary life of low-income elderly people, including those living alone. Likewise, Choi’s study [26] showed that the overall diet of elderly people living alone was inadequate in terms of quantity and quality. Since the NQ-E score of elderly people living alone reflects their dietary quality and nutritional status, appropriate measures are needed to improve their NQ-E as the NQ-E scores of elderly people living alone in this study are extremely low.

In Chung’s study [7], as a result of comparing the researcher’s nutrient intake status by classifying it into four grades according to the NQ-E score, the NQ-E upper-class group had a significantly higher intake ratio than the lower-class group, such as energy intake, vitamin A, and vitamin C, and suggested that NQ-E could be useful for nutritional evaluation of the elderly. Comparing the NQ-E in all four factors, the NQ-E of elderly people living alone was lower in all aspects than that of the Korean elderly, indicating that elderly people living alone are more exposed to the risk of malnutrition and have a poor diet than the other elderly.

As the aging population accelerates, our society also needs to improve the nutritional status of elderly people and systematically manage their diet. So, dietary welfare services should be expanded to vulnerable groups such as elderly people living alone [26]. Reasonable budgets and securing infrastructure should be included in the policy to cope with the increased number of elderly people [5], and improving the diet of elderly people can improve individuals’ quality of life and reduce social and medical costs [7].

Although this study aimed to determine the dietary life of elderly people living alone, some limitations were still observed. Collecting data on elderly people living alone was challenging, especially using the survey method, and in this study, the diet of the elderly was evaluated using a questionnaire. This study was conducted in Seoul, and future research will be directed toward investigating elderly people living alone nationwide, including those living in rural areas. The current study was conducted specifically among the elderly living alone utilizing welfare facilities in Seoul, resulting in a limited sample size due to the requirement of their cooperation. 

The revision of NQ-E for the elderly was undertaken to reflect dietary quality and behavior among 1000 Korean elderly people in 2021 [36]. Twenty-nine items of the NQ-E checklist were reduced to 23 items, and checklist items addressed balance, moderation, and practice, weighting 0.55, 0.10, and 0.35 respectively. The NQ-E of this study was 51.7, 44.9 for balance, 52.2 for moderation, and 62.1 for practice. This revised NQ-E 2021 is also available online to be applicable conveniently. In the future, NQ-E for the elderly living alone needs to be studied using the revised NQ-E 2021. However, this study is still considered significant as it evaluated the detailed dietary lifestyle and nutritional status of elderly people living alone using the NQ-E.

As a result of evaluating the dietary health of the elderly living alone who are receiving help from the local government through this study, the dietary habits of the elderly living alone were in need of improvement. The provision of meal services considering the health status of the elderly should be reviewed. The NQ-E presented in this study can be used to evaluate the dietary habits of the elderly and is expected to be used as an indicator for developing community programs for health promotion and evaluating their effectiveness.

## Figures and Tables

**Table 1:** General characteristics of the participants (*n* = 300).

Characteristics	Number (%)	Mean ± SD
Gender		
Male	26 (25.2)	
Female	77 (74.8)	
Age (years)		77.8 ± 5.2
65–74	26 (25.2)	71.2 ± 2.7
>75	77 (74.8)	80.8 ± 3.8
Educational background		
No educational experience	42 (40.7)	
Elementary school	27 (26.2)	
Middle school	17 (16.5)	
High school	9 (8.7)	
More than College or University	6 (5.8)	
No response	2 (1.9)	
Types of public welfare services		
Basic living recipient	62 (60.2)	
The secondary poor	6 (5.8)	
Not applicable	28 (27.2)	
Unknown	7 (6.8)	
Medication *		
Hypertension	55 (53.4%)	
Diabetes	26 (25.2%)	
Hyperlipidemia	19 (18.4%)	
Arthritis	23 (22.3%)	
Gastrointestinal disease	14 (13.6%)	
Anemia	3 (2.9%)	
Etc.	43 (41.7%)	

* Multiple response.

**Table 2:** Type of welfare services received from local government N(%).

Type of Service *	Male (*n* = 26)	Female (*n* = 77)	Total (*n* = 103)
Lunch box delivery	2 (7.7)	2 (2.6)	4 (3.9)
Free use of senior restaurant	17 (65.4)	38 (49.4)	55 (53.4)
Visiting service by senior life manager	26 (100.0)	76 (98.7)	102 (99.0)
Side dish delivery	7 (26.9)	11 (14.3)	18 (17.5)
Foodbank voucher	3 (11.5)	10 (13.0)	13 (12.6)
Etc.	0 (0)	1 (1.3)	1 (1.0)

* Multiple response.

**Table 3:** Food insecurity and social activity of the participants.

Variables	Male (*n* = 26)	Female (*n* = 77)	Total (*n* = 103)	χ^2^
Food insecurity				3.233
It was economically difficult; so, I often ran out of food	2 (7.7)	7 (9.1)	9 (8.7)	
It was economically difficult; so, sometimes I was short of food	5 (19.2)	19 (24.7)	24 (23.3)	
I ate enough food, but I could not eat various kinds of food	9 (34.6)	35 (45.5)	44 (42.7)	
I ate enough and different types of food as I wanted	10 (38.5)	16 (20.8)	26 (25.2)	
Social activity(leisure activity)				4.014
Not at all	5 (19.2)	11 (14.3)	16 (15.5)	
Not much	8 (30.8)	17 (22.1)	25 (24.3)	
Average	2 (7.7)	14 (18.2)	16 (15.5)	
Often	10 (38.5)	25 (32.5)	35 (34.0)	
Very often	1 (3.8)	10 (13.0)	11 (10.7)	

Values are presented as number (%). Significant difference between male and female by χ^2^ test.

**Table 4:** Nutritional status and dietary life of elderly people living alone N(%).

Variables	Male (*n* = 26)	Female (*n* = 77)	Total (*n* = 103)	χ^2^
Meal consumption				
Breakfast	19 (73.1)	61 (79.2)	80 (77.7)	1.821
Lunch	26 (100.0)	72 (93.5)	98 (95.1)	1.405
Dinner	25 (96.2)	67 (87.0)	92 (90.3)	1.751
The main reason for skipping meals ^(1)^				0.004
Lack of appetite	0 (0)	12 (15.6)	12 (11.7)	
Usual habits	1 (3.8)	6 (7.8)	7 (6.8)	
Difficulty preparing meals	3 (11.5)	3 (3.9)	6 (5.8)	
Poor digestion	0 (0)	4 (5.2)	4 (3.9)	
To save money	2 (7.7)	0 (0)	2 (1.9)	
To lose weight	0 (0)	1 (1.3)	1 (1.0)	
Etc.	3 (11.5)	3 (3.9)	6 (5.8)	
Dietary life problem ^§^				
No particular dietary issues	19 (73.1)	30 ((39.0)	49 (47.6)	
Poor appetite	2 (7.7)	15 (19.5)	17 (16.5)	
Unbalanced diet	2 (7.7)	14 (18.2)	16 (15.5)	
Eating disorders due to dental problems	2 (7.7)	13 (16.9)	15 (14.6)	
Eating salty food	1 (3.8)	10 (13.0)	11 (10.7)	
Skipping meals	1 (3.8)	5 (6.5)	6 (5.8)	
Eating irregular meals	1 (3.8)	4 (5.2)	5 (4.9)	
Eating sweets	0 (0)	5 (6.5)	5 (4.9)	
Overeating	0 (0)	1 (1.3)	1 (1.0)	
Difficulty swallowing (dysphagia)	0 (0)	1 (1.3)	1 (1.0)	
Etc.	0 (0)	4 (5.2)	4 (3.9)	

Significant difference between male and female by χ^2^ test. ^(1)^ Fisher’s exact test. * *p* < 0.05. ^§^ Not available χ^2^ value due to multiple response question item.

**Table 5:** Current status of the dietary behaviors of elderly people living alone N(%).

Variables	Male (*n* = 26)	Female (*n* = 77)	Total (*n* = 103)	*p*
Number of side dishes of vegetables per meal (excluding Kimchi)				0.752
None	5 (19.2)	9 (11.7)	14 (13.6)	
One	8 (30.8)	22 (28.6)	30 (29.1)	
Two	7 (26.9)	27 (35.1)	34 (33.0)	
Three	6 (23.1)	16 (20.8)	22 (21.4)	
Four or more	0 (0)	3 (3.9)	3 (2.9)	
Water intake frequency				0.117
Rarely	0 (0)	1 (1.3)	1 (1.0)	
1–2 times a day	3 (11.5)	13 (16.9)	16 (15.5)	
3–5 times a day	8 (30.8)	39 (50.6)	47 (45.6)	
6–7 times a day	7 (26.9)	15 (19.5)	22 (21.4)	
More than 8 times a day	8 (30.8)	9 (11.7)	17 (16.5)	
Number of meals per day				0.352
One	1 (3.8)	3 (3.9)	4 (3.9)	
Two	6 (11.5)	29 (37.7)	35 (34.0)	
Three or more	19 (73.1)	45 (58.4)	64 (62.1)	
Number of snacks per day				0.634
None	21 (80.8)	52 (67.5)	73 (70.9)	
One	4 (15.4)	20 (26.0)	24 (23.3)	
Two	1 (3.8)	4 (5.2)	5 (4.9)	
More than 3 times	0 (0)	1 (1.3)	1 (1.0)	
Number of times eating alone				0.307
Rarely	1 (3.8)	0 (0)	1 (1.0)	
4–6 times a week	0 (0)	3 (3.9)	3 (2.9)	
1 times a day	7 (26.9)	16 (20.8)	23 (22.3)	
2 times a day	15 (57.7)	41 (53.2)	56 (54.4)	
3 times a day	3 (11.5)	17 (22.1)	20 (19.4)	
Efforts to eat a healthy diet				0.777
Rarely	3 (11.5)	5 (6.5)	8 (7.8)	
Lack of effort	2 (7.7)	12 (15.6)	14 (13.6)	
Average	4 (15.4)	11 (14.3)	15 (14.6)	
Trying	15 (57.7)	44 (57.1)	59 (57.3)	
Trying very hard	2 (7.7)	5 (6.5)	7 (6.8)	
Chewing ability				0.496
Not at all uncomfortable	4 (15.4)	16 (20.8)	20 (19.4)	
Not uncomfortable	7 (26.9)	16 (20.8)	23 (22.3)	
Average	3 (11.5)	6 (7.8)	9 (8.7)	
Uncomfortable	10 (38.5)	23 (29.9)	33 (32.0)	
Very uncomfortable	2 (7.7)	16 (20.8)	18 (17.5)	
Washing hands				0.642
Not enough	2 (7.7)	4 (5.2)	6 (5.8)	
Average	0 (0)	2 (2.6)	2 (1.9)	
Often	9 (34.6)	19 (24.7)	28 (27.2)	
Aways	15 (57.7)	52 (67.5)	67 (65.)	
Exercise				0.163
Rarely	3 (11.5)	16 (20.8)	19 (18.4)	
<30 min	6 (23.1)	16 (20.8)	22 (21.4)	
30 min–1 h	7 (26.9)	31 (40.3)	38 (36.9)	
1–2 h	5 (19.2)	10 (13.0)	15 (14.6)	
>2 h	5 (19.2)	4 (5.2)	9 (8.7)	
Depression				0.461
Rarely	6 (23.1)	14 (18.2)	20 (19.4)	
Not often	6 (23.1)	12 (15.6)	18 (17.5)	
Average	4 (15.4)	11 (14.3)	15 (14.6)	
Often	8 (30.8)	22 (28.6)	30 (27.1)	
Very often	2 (7.7)	18 (23.4)	20 (19.4)	
Self-health assessment level				0.147
Not healthy at all	0 (0)	6 (7.8)	6 (5.8)	
Not healthy	5 (19.2)	29 (37.7)	34 (33.0)	
Average	4 (15.4)	7 (9.1)	11 (10.7)	
Healthy	16 (61.5)	31 (40.3)	47 (45.6)	
Very healthy	1 (3.8)	4 (5.2)	5 (4.9)	

Significant difference between male and female by Fisher’s exact test.

**Table 6:** Comparison of NQ-E scores on elderly people living alone (mean±SD) N(%).

Variables *			Weighted Score for Each Evaluation Item	Male (*n* = 26)	Female (*n* = 77)	Total (*n* = 103)	t
Factor	Balance	Drink milk or dairy products	11.96 ± 10.44				
		Fruit intake	18.77 ± 15.64	39.05 ± 24.78	37.60 ± 21.34	37.96 ± 22.15	0.288
		Number of snacks	2.26 ± 3.91				
		Water intake	4.97 ± 2.03				
	Moderation	Sweet food	29.95 ± 7.64	60.94 ± 15.82	65.24 ± 15.10	64.16 ± 15.32	−1.240
		Sweetened beverages	20.10 ± 12.78				
		Ramen	14.11 ± 2.85				
	Diversity	Eggs	5.60 ± 4.66	34.97 ± 11.15	35.17 ± 12.77	35.12 ± 12.33	−0.071
		Fish or shellfish	2.45 ± 3.64				
		Eating alone	5.99 ± 4.33				
		Vegetable	11.02 ± 6.70				
		Beans or soy products	5.87 ± 4.33				
		Number of meals	4.19 ± 1.50				
	Dietary behavior	The degree of discomfort in chewing food	11.02 ± 8.06	59.89 ± 8.82	49.88 ± 10.41	50.14 ± 10.00	1.904
		Awareness of health	10.59 ± 5.51				
		The usual degree of depression	10.19 ± 7.52				
		Washing hands before eating	8.70 ± 1.98				
		Exercise time per day	5.30 ± 3.58				
		The effort of healthy eating	8.46 ± 3.70				
Nutrition Quotients *				50.89 ± 8.82	49.88 ± 10.41	50.14 ± 10.00	0.443
Result				Monitoring needed	Monitoring needed	Monitoring needed	

Significant difference between male and female by independent *t*-test. * The nutritional quotient criteria for elderly people is 62 points. Nutrition quotients were calculated by Balance ×0.2 + moderation ×0.3 + diversity ×0.3 + dietary behavior ×0.2.

**Table 7:** NQ-E score distribution by the four-grade criteria ^(1)^ N(%).

Variables	Grade Criteria			
	High	Medium-High	Medium-Low	Low
NQ-E ^(2)^	9 (8.7)	15 (14.6)	23 (22.3)	56 (54.4)
Balance ^(3)^	28 (27.2)	16 (15.5)	29 (28.2)	30 (29.1)
Moderation ^(4)^	0 (0)	37 (35.9)	12 (11.7)	54 (52.4)
Diversity ^(5)^	2 (1.9)	11 (10.7)	23 (22.3)	67 (65.0)
Dietary behavior ^(6)^	28 (27.2)	19 (18.4)	28 (27.2)	28 (28.2)

Values are presented as number. *n* = 103. ^(1)^ Referred from Chung et al.’s study [7] of nutrition quotients for the elderly; High: 75% ≤ NQ-E percentile ≤ 100%, Medium-high: 50% < NQ-E percentile < 75%, Medium-low: 25% ≤ NQ-E percentile < 50%, Low: 0% ≤ NQ-E percentile < 25%. ^(2)^ High: 63.5–100, medium-high: 57.6–63.4, medium-low: 51.9–57.5, low: 0–51.8. ^(3)^ High: 55.2–100, medium-high: 41.6–55.1, medium-low: 25.8–41.5, low: 0–25.7. ^(4)^ High: 91.5–100, medium-high: 76.3–91.4, medium-low: 67.8–76.2, low: 0–67.7. ^(5)^ High: 60.0–100, medium-high: 50.5–59.9, medium-low: 40.3–50.4, low: 0–40.2. ^(6)^ High: 64.9–100, medium-high: 55.1–64.8, medium-low: 42.2–55.0, low: 0–45.1.

## Data Availability

Not applicable.
